# Blind-Ending Esophageal Fistula Complicating Eosinophilic Esophagitis: Case Report and Up-to-Date Strategy from Diagnosis to Therapy

**DOI:** 10.3390/diagnostics16091374

**Published:** 2026-04-30

**Authors:** Anthony Rasuceanu, Adrian Constantin, Florin Achim, Alex-Claudiu Moraru, Alexandru Rotariu, Andreea-Gabriela Manole, Petre Hoara, Roxana-Elena Stefan, Madalina-Georgiana Mitrea-Tocitu, Cristian Rosianu, Anca Evsei-Seceleanu, Dragos-Viorel Scripcariu, Dragos Predescu

**Affiliations:** 1 Doctoral School, Carol Davila University of Medicine and Pharmacy, 050474 Bucharest, Romania; rasuceanu.anthony@gmail.com (A.R.); alex.claudiu.moraru@gmail.com (A.-C.M.); alexandru.rotariu95@yahoo.com (A.R.); roxana-elena.stefan@drd.umfcd.ro (R.-E.S.); mitrea.madalina@ymail.com (M.-G.M.-T.); 2 Faculty of Medicine, Carol Davila University of Medicine and Pharmacy, 050474 Bucharest, Romania; dradiconstantin@yahoo.com (A.C.); petre_hoara@yahoo.com (P.H.); anca.evsei@umfcd.ro (A.E.-S.); drpredescu@yahoo.com (D.P.); 3 Department of Esophageal and General Surgery, Sf. Maria Clinical Hospital, 011192 Bucharest, Romania; manoleandreeagabriela@gmail.com; 4 Department of Gastroenterology, Sf. Maria Clinical Hospital, 011192 Bucharest, Romania; rosianu_cristian@yahoo.com; 5 Department of Pathology, Sf. Maria Clinical Hospital, 011192 Bucharest, Romania; 6 Faculty of Medicine, Grigore T. Popa University of Medicine and Pharmacy, 700115 Iasi, Romania; 7 First Oncological Surgery Unit, Regional Institute of Oncology, 700483 Iasi, Romania

**Keywords:** eosinophilic esophagitis, esophageal fistula, endoscopic treatment, elimination diet

## Abstract

**Background and Clinical Significance:** Eosinophilic esophagitis (EoE) is a chronic, immune-mediated inflammatory disorder characterized by esophageal dysfunction and dense eosinophilic infiltration. EoE frequently evolves into a fibrostenotic phenotype, in which uncontrolled inflammation drives progressive tissue remodeling. This evolution increases the risk of complex structural complications—most commonly fixed rings and strictures, and, in rare advanced cases, deep mural injury or fistulization—substantially increasing both diagnostic and therapeutic complexity. **Case Presentation:** This report describes an uncommon presentation of EoE complicated by a blind-ending esophageal fistula, managed successfully through a multidisciplinary strategy integrating pharmacologic therapy, dietary modification, and endoscopic intervention. **Conclusions:** Nutritional support through gastrostomy, combined with multidisciplinary medical and endoscopic management, can lead to favorable outcomes in EoE complicated by esophageal fistula. Early recognition and individualized management are essential to optimize outcomes.

## 1. Introduction and Clinical Significance

Eosinophilic esophagitis (EoE) is a chronic immune-mediated inflammatory disorder of the esophagus, defined by symptoms of esophageal dysfunction and a dense eosinophilic infiltrate in the mucosa [1]. Although cases of esophageal eosinophilia were described in the 1960s and 1970s, EoE was recognized as a distinct clinical entity only after a 1993 case series identified patients with esophageal eosinophilia in the absence of acid reflux [2]. Since then, its incidence has risen steadily to 5–10 new cases per 100,000 individuals annually, with prevalence estimates approaching ~60 per 100,000 in some regions [3]. The disease predominantly affects Caucasian males (M:F ratio ~3:1) and is strongly associated with atopic conditions, including allergic rhinitis, asthma and eczema [4].

EoE has become the second most common cause of chronic esophagitis after gastroesophageal reflux disease (GERD) [5] and the leading cause of esophageal food impaction in young patients presenting to emergency departments [6].

Clinical manifestations vary by age: young children may present with feeding difficulties, vomiting or failure to thrive, whereas adolescents and adults typically report intermittent dysphagia (present in ~70% of cases) [7] and recurrent food impaction (33–54% of cases) [8].

Diagnosis relies on the integration of clinical symptoms, characteristic endoscopic findings (concentric rings, longitudinal grooves, whitish exudates, edema) and histologic evidence of ≥15 eosinophils per high-power field (HPF), after exclusion of alternative causes of esophageal eosinophilia [1]. Treatment aims to achieve and maintain clinical and histologic remission while preventing fibrostenotic complications. Current therapeutic strategies include dietary elimination, anti-inflammatory pharmacologic therapy, and endoscopic intervention [9].

*Clinical Significance:* With rising incidence and increasing recognition of complex presentations, EoE has become a significant challenge in upper gastrointestinal practice [10]. Although most patients have a favorable prognosis, atypical or complicated forms (such as those involving fistulas, abscesses or pseudodiverticula) pose substantial diagnostic and therapeutic difficulties [11].

This article reports a rare case of EoE complicated by a blind-ending esophageal fistula and provides a narrative review of current evidence, emphasizing therapeutic options and their clinical implications.

## 2. Case Presentation and Literature Review—Search Strategy

This study integrates a detailed case report with a narrative review of the literature on eosinophilic esophagitis and its uncommon complications.

### 2.1. Case Report Design

The clinical case was documented at the Department of General and Esophageal Surgery, Sf. Maria Clinical Hospital, Bucharest, Romania. All diagnostic and therapeutic procedures—including upper endoscopy, computed tomography (CT), and histopathologic evaluation—were performed as part of routine clinical care. Clinical data, imaging studies, and histopathologic findings were retrospectively extracted from the patient’s medical records, anonymized and analyzed descriptively. Written informed consent was obtained from the patient for publication of this case report and accompanying images.

### 2.2. Literature Review—Search Strategy

**Figure 1:**
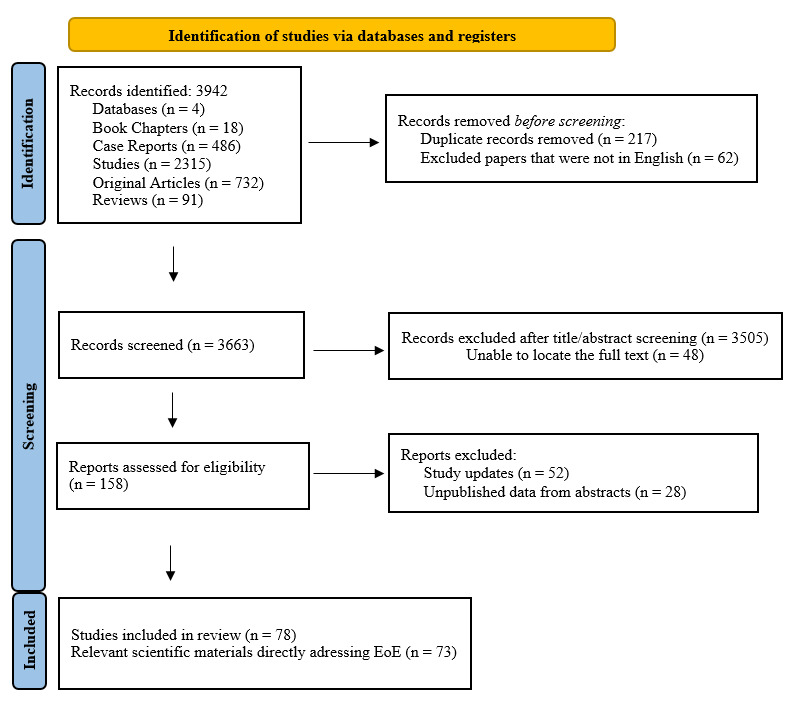
PRISMA flow diagram illustrating the study selection process for the narrative review. A total of 3942 records were identified through database searching (PubMed, Cochrane Library, Embase, and MEDLINE). After removal of duplicate records (*n* = 217) and non-English publications (*n* = 62), 3663 records underwent title and abstract screening. Following screening, 158 full-text reports were assessed for eligibility, while 48 reports were not retrieved. Among the assessed full-text reports, 52 study updates and 28 unpublished abstract-only records were excluded. Ultimately, 78 studies were included in the final qualitative synthesis.

A narrative literature review was performed to contextualize the presented case and synthesize current evidence on the diagnosis, histopathologic criteria, and therapeutic management of EoE. Electronic searches were conducted in PubMed, Cochrane Library, Embase (Excerpta Medica Database), and MEDLINE Complete (EBSCO) covering the last 25 years, using the following terms: “eosinophilic esophagitis,” “fibrostenotic,” “fistula,” “treatment,” “diet,” “steroids,” and “*dupilumab*.” The initial search yielded 3942 records, including (Figure 1): book chapters (18), case reports (486), original articles (732), studies (2315), and reviews (91).

To refine the search toward EoE-specific complications, advanced filters and additional terms—“histopathology,” “complications,” “endoscopic management,” “topical corticosteroid treatment,” and “dietary elimination”—were applied using Boolean operators AND and OR. Ultimately, 78 publications directly addressing EoE and its complications were included after full-text eligibility assessment. Additional relevant articles from the initial search were included through manual cross-referencing. Two independent reviewers (A.R. and D.P.) assessed the studies for eligibility, prioritizing peer-reviewed publications in English from high-impact journals. Disagreements were resolved through discussion, with a third reviewer (A.C.) providing adjudication when necessary. Studies available only as abstracts, unpublished data and non-English publications were excluded from the final analysis.

### 2.3. Case Presentation

Our department is a tertiary referral center for general and esophageal surgery, with experience in the management of complex esophageal pathology. Over the past two decades, seven cases of eosinophilic esophagitis (EoE) have been diagnosed and managed at our institution. Among these, we report a case complicated by a blind-ending esophageal fistula, selected for its favorable outcome and multidisciplinary management.

A 22-year-old patient with a history of bronchial asthma, allergic rhinitis and nasal polyposis, presented to the emergency department with progressive dysphagia for solid foods and anterior chest pain radiating posteriorly, exacerbated by swallowing and after meals. Symptoms started six days before hospital admission.

**Figure 2:**
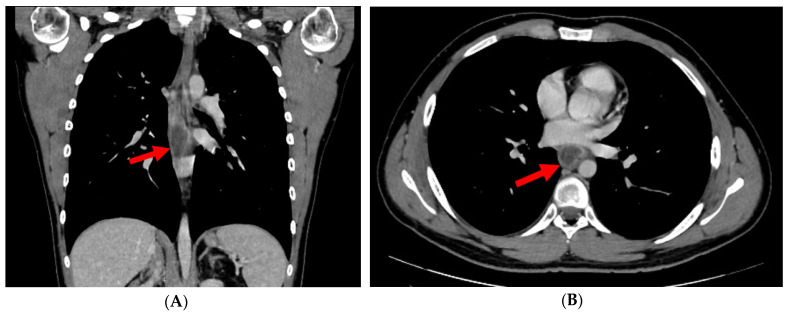
(**A**,**B**) Contrast-enhanced CT scan performed during the venous phase of contrast circulation, in axial (**A**) and coronal planes (**B**): Localized intramural collection located in the right anterolateral aspect of the middle thoracic esophagus, with modified fluid densities (34 Hounsfield Units) and slight mass effect on the esophageal lumen, which is displaced medially (red arrows).

On examination, the patient was afebrile and hemodynamically stable, with normal cardiopulmonary and abdominal findings. Laboratory studies showed leukocytosis with neutrophilia and markedly elevated C-reactive protein (150 mg/L). Computed tomography (CT) of the thorax and upper abdomen demonstrated inflammatory thickening of the mid-thoracic esophageal wall and a well-defined parafluid intramural collection along the right anterolateral aspect (Figure 2A,B, red arrows).

**Figure 3:**
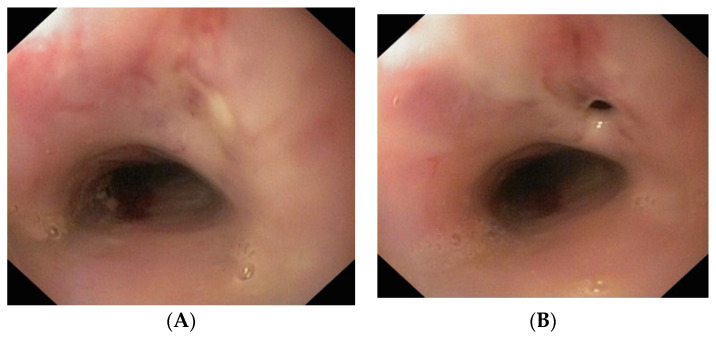
(**A**) Endoscopic image of EoE with an esophageal fistula covered with purulent material. (**B**) Endoscopic image of EoE with an esophageal fistula after removal of the purulent material.

Upper digestive endoscopy revealed diffusely modified esophageal mucosa and approximately 32 cm from the incisors, a fistulous opening draining purulent material. The mucosa was friable, prompting superficial biopsies (Figure 3A,B). A 3–4 cm hiatal hernia was also noted, while the stomach and duodenum appeared normal.

Based on clinical, endoscopic findings—EREFS score 5—moderate severity (edema—1, rings—1, exudates—2, furrows—0, stricture—1)—and imaging—EoE was considered the most likely diagnosis. Differential diagnoses—including GERD, Crohn’s disease, nonsteroidal anti-inflammatory drug (NSAID)-induced injury, Marfan syndrome type II and hyper-IgE syndrome were excluded.

**Figure 4:**
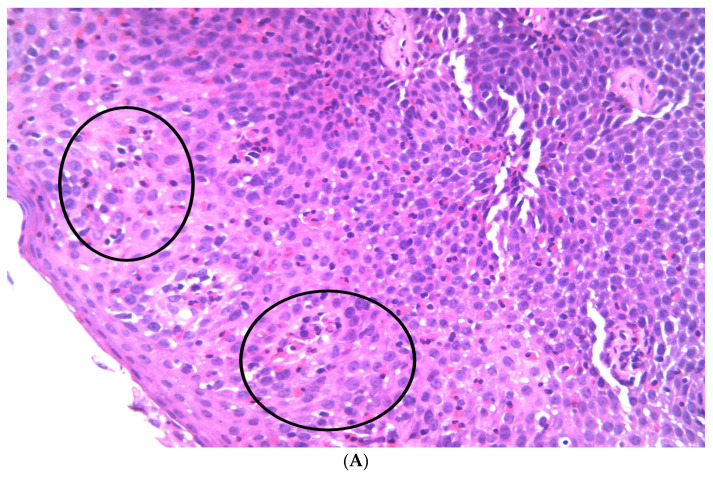

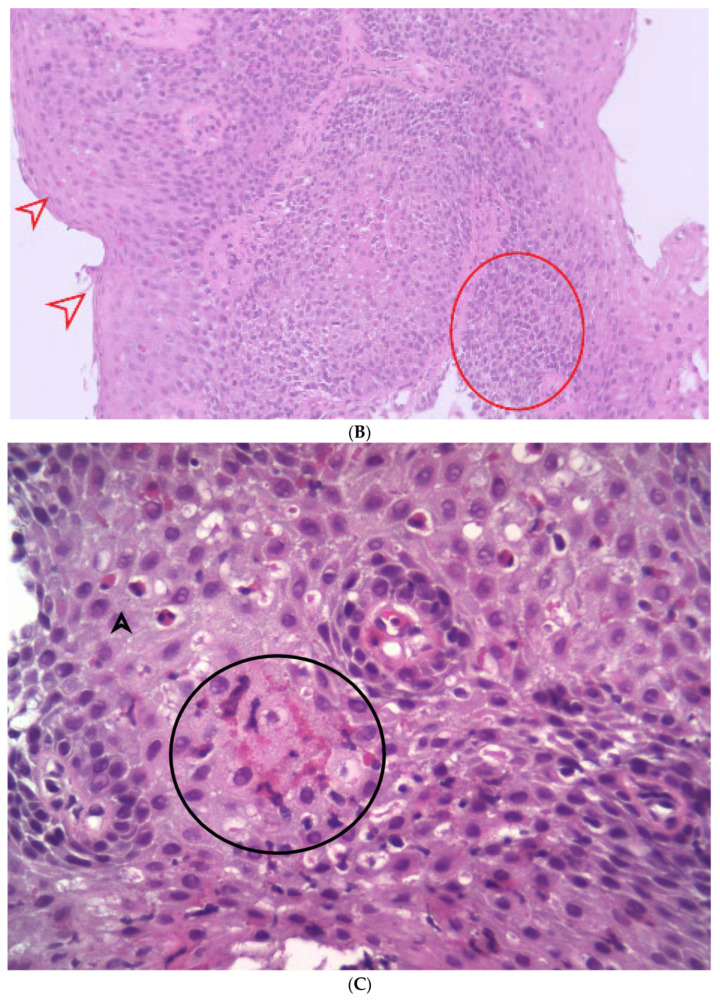
(**A**). Hematoxylin–Eosin, 400× magnification: Esophageal squamous epithelium showing marked intraepithelial eosinophilic infiltration (black circles). (**B**). Hematoxylin–Eosin, 200× magnification: Esophageal mucosa with extreme basal cell hyperplasia (red circle) and elongation of lamina propria papillae, associated with superficial eosinophilic infiltration (red arrows). (**C**). Hematoxylin–Eosin, 600× magnification: Eosinophil degranulation within the squamous epithelium (black circle), with extracellular eosinophilic granules dispersed among epithelial cells; non-degranulated eosinophils are also present (black arrow).

The pathological evaluation revealed an esophageal squamous mucosa with an eosinophilic infiltrate exceeding 15 eosinophils per high-power field (HPF) (Figure 4A, black circles), with a peak density of 45 eosinophils/HPF, eosinophils concentrated in the surface epithelium (Figure 4B, red arrows), extreme basal hyperplasia (Figure 4B, red circle) and eosinophil degranulation (Figure 4C, black circle). A diagnosis of eosinophilic esophagitis was confirmed.

Treatment was initiated with *omeprazole* (40 mg twice daily), topical corticosteroid (*fluticasone*) (440 µg twice daily), broad-spectrum antibiotics, total fasting with saliva drainage, and parenteral nutritional support. Although proton pump inhibitors (PPIs) are typically used as first-line therapy, corticosteroids were added in this case due to the presence of an esophageal fistula, a complication associated with more severe inflammatory activity.

**Figure 5:**
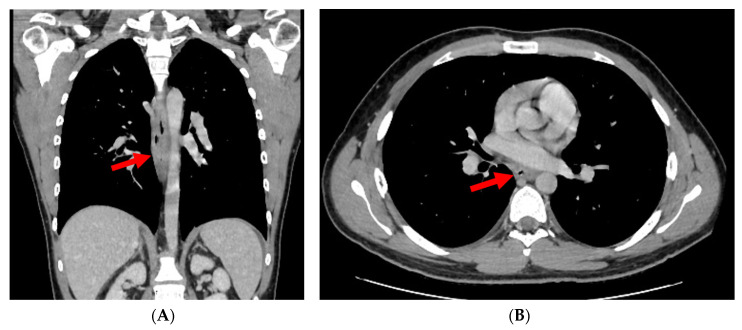
(**A**,**B**). Contrast-enhanced CT scan performed during the venous phase of contrast circulation, in axial and coronal planes: complete regression of the intramural collection visualised in the middle thoracic esophagus (red arrows).

After seven days, repeat computed tomography imaging showed complete resolution of the intramural collection (Figure 5A,B, red arrows).

**Figure 6:**
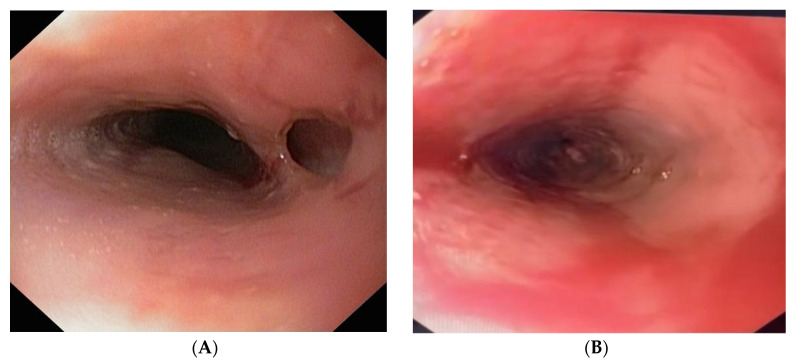
(**A**) Endoscopic image of active EoE, with a blind esophageal fistula measuring approximately 3–4 mm. (**B**) Endoscopic image of EoE in an active flare (>40 eosinophils/HPF).

On day eight after admission, follow-up endoscopy demonstrated a fistulous tract at 30–32 cm from the incisors without mediastinal communication, along with diffuse mucosal edema, hyperemia and friability (Figure 6A,B). Superficial biopsies were taken. Given impaired esophageal function, a 20-Fr percutaneous endoscopic gastrostomy (PEG) tube was placed to ensure enteral nutrition. The patient was kept on complete fasting and was instructed to avoid swallowing saliva and received six-food elimination diet via gastrostomy given the concomitant diagnosis of a 3–4 cm hiatal hernia with possible gastroesophageal reflux.

**Figure 7:**
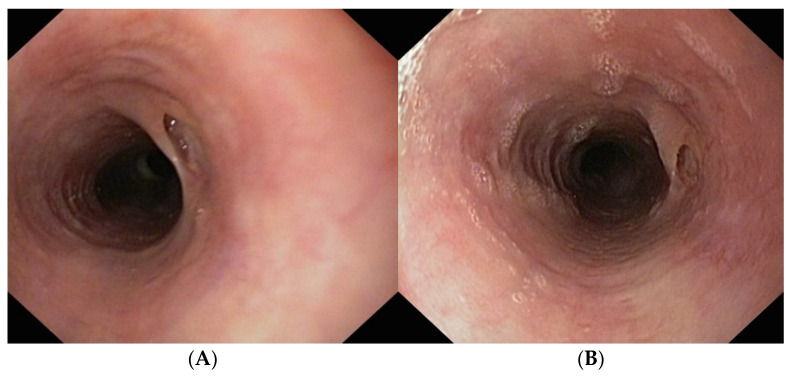
(**A**) Endoscopic image of EoE during treatment (>15 eosinophils/HPF, peak density of 25 eosinophils/HPF). (**B**) Endoscopic image of EoE in complete remission (1 eosinophils/HPF).

Follow-up endoscopy four weeks after PEG placement revealed marked improvement in mucosal appearance and a 3–4 mm blind-ending fistula in remission. (Figure 7A) The esophagus showed mild narrowing and a trachealized appearance in the lower third but allowed passage of the endoscope.

Follow-up endoscopy seven weeks after PEG placement, endoscopy identified a 1-cm blind-ending pseudodiverticulum at the site of the previous fistulous tract, with otherwise normal mucosa. (Figure 7B). The PEG tube was removed, oral feeding was resumed, and treatment with corticosteroid and antisecretory agents was continued. Biopsies taken demonstrated only 1 eosinophil per high-power field.

The patient remained under clinical observation, in collaboration with the gastroenterology department, and with periodic endoscopic and histologic assessments during the gradual reintroduction of the six eliminated allergens.

A focused literature review further outlines current histopathologic criteria, key considerations in differentiating EoE from gastroesophageal reflux disease, and recent therapeutic advances including proton pump inhibitors, topical corticosteroids, endoscopic dilation and emerging biologic agents.

## 3. Discussion

An essential component in the management of patients with EoE is early recognition of the disease achieved through upper gastrointestinal endoscopy with biopsy sampling at symptom onset as reported by the patient. Histopathological evaluation of esophageal mucosal specimens enables timely confirmation of eosinophilic esophagitis.

In the presented case, early upper endoscopy with biopsy was essential not only for confirming EoE, but also for clarifying the cause of an unusual structural complication, namely a blind-ending esophageal fistula.

*The histopathologic features of EoE include, as a significant criterion, an eosinophilic count of over 15 eosinophils/HPF.* Other key histologic features include eosinophilic microabscesses in the esophageal squamous epithelium, eosinophil surface layering, and eosinophil degranulation. In other cases, a marked *basal hyperplasia* can be observed, and sometimes *lamina propria* fibrosis may be present [11]. In our patient, histopathologic examination revealed marked eosinophilic infiltration, with a peak of 45 eosinophils per high-power field, supporting active and severe mucosal inflammation in the context of a structural complication.

In this context, detailed histopathological evaluation becomes essential for disease staging and identification of remodeling components, and the *Eosinophilic Esophagitis Histology Scoring System (EoEHSS)* provides a standardized framework for quantification [12,13].

The EoEHSS was proposed and validated by Collins et al. in 2016 with the aim of improving the histopathologic evaluation of eosinophilic esophagitis beyond peak eosinophil count alone [13]. The EoEHSS includes eight features of esophageal biopsy specimens (Table 1). These were defined as follows: eosinophilic inflammation, basal hyperplasia, eosinophil abscess, eosinophil surface layering, dilated intercellular spaces, surface epithelial alteration, dyskeratotic epithelial cells, and *lamina propria* fibrosis. Every criterion was assigned a score for severity and extent [12]. The main idea was to achieve a better diagnosis using more parameters than the usual count of eosinophils. Although it is a structured scoring system with growing uptake, further validation across settings and harmonization of reporting remain important [14].

Each histologic feature included in the EoEHSS is scored separately for severity (grade) and extent (stage) using a semi-quantitative scale ranging from 0 (absent) to 3 (most severe or extensive), as originally described by Collins et al. [13]. The grade reflects the intensity of the histologic abnormality within affected areas, whereas the stage describes the proportion of the epithelium involved [12]. Importantly, the EoEHSS does not generate a single composite score; instead, individual feature scores are interpreted in a multidimensional manner, allowing distinction between active inflammatory disease and chronic fibrostenotic remodeling. This approach provides a more comprehensive assessment of disease activity and tissue remodeling than peak eosinophil count alone, particularly in patients with discordant symptoms or established structural complications [12,13].

Because the EoEHSS captures both active inflammation and fibrosis, it helps contextualize the evolution of EoE toward fibrostenotic disease [12,15,16,17].

The progression of untreated or poorly controlled eosinophilic esophagitis leads, over time, to the development of a fibrostenotic phenotype, characterized by esophageal rings, strictures and progressive narrowing of the lumen [18]. These changes reflect fibrous remodeling of the esophageal wall, with loss of elasticity and the development of persistent dysphagia, especially for solid foods [19]. Once established, these structural changes become partially irreversible and cannot be corrected by anti-inflammatory therapy alone, justifying the need for mechanical interventions [16]. In rare advanced cases, chronic inflammation may extend beyond the mucosa and lead to deep mural injury, which may explain the development of the blind-ending fistula observed in the reported case.

Although histopathologic diagnosis is generally reliable, there is always the challenge of differentiating it from gastroesophageal reflux disease (GERD), since the current therapeutic approaches are different [20]. There are a few differences between the two entities. GERD usually shows hyperplasia of *lamina propria* papillae, mild basal hyperplasia, and a mild eosinophilic count (usually <10 eosinophils/HPF). These histopathological features are not pathognomonic; therefore, each case must be reported in correlation with the clinical and endoscopic aspects. Another helpful feature is the higher distribution of eosinophils in the distal esophagus in GERD and the higher distribution of eosinophils in the proximal esophagus in EoE. Sometimes, there can be overlap between the two diseases, so it is advisable that the clinician obtain biopsies primarily from the proximal and mid esophagus to help the pathologist provide the best diagnosis. In the present case, the diagnosis of EoE was supported by clinical presentation, endoscopic findings, and histopathologic confirmation, while other causes of esophageal injury were excluded.

Beyond these morphological overlaps, the distinction between GERD and eosinophilic esophagitis reflects fundamentally different pathogenic mechanisms, which translate into distinct patterns of disease progression and response to therapy [21,22]. In EoE, persistent immune-mediated inflammation drives progressive tissue remodeling and fibrotic narrowing of the esophageal lumen, thereby creating the structural substrate for fibrostenotic complications that require mechanical intervention [23,24]. In our patient, this process likely progressed to focal mural disruption, resulting in fistula formation.

Another key objective in the management of eosinophilic esophagitis is the prompt initiation of disease-specific pharmacologic therapy aimed at reducing mucosal inflammation. In the present case, early endoscopy raised suspicion of EoE and biopsies were obtained according to protocol, with histopathologic examination confirming the diagnosis and demonstrating a marked eosinophilic infiltrate (peak 45 eosinophils/HPF) according to the EoEHSS. The presence of a fistulous tract and intramural collection indicated severe inflammatory disease, supporting early initiation of combined conservative therapy.

Therapeutic options for eosinophilic esophagitis are relatively limited and applied in a stepwise manner, ranging from pharmacological, dietary and endoscopic therapies to surgical approaches in selected cases.

The primary goal of pharmacological treatment in eosinophilic esophagitis is to control eosinophilic inflammation, prevent fibrous remodeling, and reduce the risk of fibrostenotic complications [25]. The choice of drug therapy is based on disease severity, clinical phenotype, and response to previous treatments, in a stepwise approach that includes proton pump inhibitors and topical corticosteroids as first-line options, and in refractory or severe cases, biologic therapies [17,26]. In this context, topical oral corticosteroid therapy is the standard of care for inducing remission in eosinophilic esophagitis and has a central role in preventing fibrostenotic complications [27,28].

Corticosteroids such as *budesonide* (oral suspension or orodispersible tablets) and *fluticasone* (inhaled aerosol) rapidly reduce eosinophilic esophageal inflammation, improving symptoms of dysphagia and healing mucosal lesions in over 50–70% of patients [29,30]. In our patient, treatment with topical corticosteroids combined with proton pump inhibitors resulted in rapid clinical and radiologic improvement, including resolution of the intramural collection and progressive closure of the fistulous tract.

In addition, long-term studies indicate that sustained topical treatment can partially reverse fibrous remodeling [26,27]. Topical corticosteroids, such as budesonide and fluticasone, effectively reduce eosinophilic inflammation, improve dysphagia, and promote mucosal healing, thereby preventing or limiting fibrostenotic complications [29,31]. Long-term studies, including a retrospective study by Greuter et al., have shown that continuous therapy may reduce esophageal narrowing and maintain symptom control [26]. In addition, topical therapy has a favorable safety profile due to minimal systemic absorption, making it suitable for long-term use [26,27,31]. In our patient, topical corticosteroids were combined with PPI therapy because the fistula was considered a marker of severe inflammatory activity. This approach was followed by rapid radiologic resolution of the intramural collection and progressive endoscopic healing of the fistulous tract.

PPIs represent a first-line therapeutic option in EoE, being useful both for identifying PPI-responsive disease and as treatment due to their direct anti-inflammatory effects, independent of acid suppression [28]. Administered in twice-daily dosing for at least 8 weeks, PPIs induce histologic remission in approximately 50% of patients, with efficacy comparable to topical corticosteroids in many cases [29,32]. The efficacy of PPIs in EoE is not limited to acid suppression: molecular studies have shown that omeprazole and other PPIs directly inhibit Th2-mediated inflammatory pathways in the esophageal mucosa—for example, they block the binding of the transcription factor STAT6 to the promoter of the eotaxin-3 gene, reducing the production of this chemokine involved in eosinophil recruitment [33]. PPIs may also improve the barrier function of the esophageal epithelium and normalize the expression of epithelial genes altered in EoE, thereby contributing to the reduction in local inflammation [31,33]. It is essential that physicians explain this anti-inflammatory mechanism of PPIs to patients with EoE, as many people associate PPIs strictly with GERD; however, in reality, the benefit in EoE occurs even in patients without reflux symptoms, through the immunomodulatory effects mentioned above [31,32]. In practice, PPI treatment (e.g., *omeprazole* 20–40 mg twice daily) for at least 2 months is recommended in all newly diagnosed patients, given the high safety profile and the chance of remission. Inducing and maintaining EoE remission with PPIs may prevent the progression of inflammation to fibrosis and reduce the risk of long-term strictures, similar to other anti-inflammatory therapies [28,30,32]. PPIs can be continued as maintenance therapy at the minimum effective dose, especially in patients who are initially responsive and in whom it is desired to avoid corticosteroid therapy [29].

In the presented patient, therapy with fluticasone 440 µg twice daily and a PPI 40 mg twice daily proved effective. At the 4-week assessment, remission of the esophagitis lesions was observed, including resolution of the fistulous tract.

Biologic therapy should be considered in patients with EoE refractory to conventional treatment or with a pronounced fibrostenotic phenotype [34]. Dupilumab, the first approved biologic agent (2022), is a monoclonal antibody targeting the IL-4/IL-13 receptor pathway, central to Th2-mediated inflammation [35]. Phase III data have shown that weekly administration induces histologic remission in a substantial proportion of patients and significantly improves dysphagia symptoms, with sustained efficacy and a favorable safety profile over time [36,37]. By reducing eosinophilic inflammation and IL-13-mediated fibrogenesis, dupilumab may also improve esophageal distensibility, as assessed by EndoFLIP—Endoluminal Functional Lumen Imaging Probe, even in the absence of mechanical dilation [36].

This biologic agent is particularly indicated in patients with inadequate response to topical corticosteroids or with persistent or recurrent disease despite ongoing maintenance therapy, as well as in those with EoE and multiple atopic comorbidities (asthma, atopic dermatitis), a context in which *dupilumab* brings concomitant benefits to all of the patient’s allergic diseases [36].

Current guidelines recommend dupilumab as a step-up therapy after failure of conventional treatments, although earlier use may be considered in selected patients with severe disease or relevant atopic comorbidities [31]. In addition to dupilumab, several targeted biologic therapies are under investigation in EoE [38]. Strategies targeting IL-5, a key cytokine in eosinophil recruitment, such as mepolizumab and reslizumab, have demonstrated reductions in eosinophilic infiltration but limited clinical benefit [39,40,41]. Other approaches include targeting the IL-5 receptor (benralizumab) and IL-13 (cendakimab), with evidence of anti-inflammatory and antifibrotic effects, although further data are needed. Targeting the Siglec-8 receptor with agents such as lirentelimab has shown the ability to induce eosinophil apoptosis and inhibit mast cell activation, suggesting potential therapeutic benefit [42,43,44]. Overall, emerging biologic therapies may provide personalized treatment options for refractory EoE in the future [44,45].

Janus kinase (JAK) inhibitors have emerged as potential therapeutic options in EoE, although their role remains insufficiently defined [46,47]. EoE is a Th2-mediated disease driven by cytokines such as IL-4, IL-5, and IL-13, which signal through the JAK-STAT pathway, providing a strong rationale for JAK inhibition [48]. Evidence from other immune-mediated diseases, including ulcerative colitis and Crohn’s disease, supports their ability to induce and maintain remission by targeting multiple cytokine pathways [49]. Early reports, including case-based data, suggest potential benefit in eosinophilic gastrointestinal disorders with agents such as tofacitinib and upadacitinib [46,50]. However, current evidence remains limited, and JAK inhibitors are still considered investigational in EoE, requiring further clinical validation [51].

Currently, dietary intervention targeting food antigens plays an important role in the management of EoE and may be used either alone or in combination with pharmacologic therapy [52,53].

The most commonly used is *the six-food elimination diet* (SFED), which involves the exclusion of dairy, wheat (gluten), eggs, soy, tree nuts/seeds, and fish/seafood for a minimum of 6 weeks, followed by endoscopic evaluation and gradual reintroduction of each trigger food [54].

SFED has been shown to be an effective treatment in both children and adults, achieving histologic remission in multiple studies and meta-analyses [52]. Clinical studies have demonstrated significant reductions in eosinophilic inflammation and sustained remission with continued elimination of trigger foods [54,55,56]. Reintroduction of eliminated foods is associated with disease relapse, supporting the role of food antigens in EoE pathogenesis [56]. Cow’s milk is the most common dietary trigger, followed by gluten-containing grains and eggs [57].

To reduce dietary restrictions, step-up elimination strategies have been proposed, starting with less restrictive approaches such as two-food (2-FED) or four-food (4-FED) elimination diets and escalating to the six-food elimination diet (SFED) in non-responders [57]. These strategies suggest that most cases of EoE are driven by a limited number of food triggers [58].

An alternative to empiric elimination diets is a test-directed approach based on allergologic testing; however, its efficacy is limited, particularly in adults [56,59]. Studies have shown lower remission rates compared with empiric diets, and current guidelines do not recommend its routine use [52,59]. Beyond symptom control, elimination diets play an important role in preventing fibrotic progression by reducing eosinophilic inflammation and promoting mucosal healing [60,61].

The efficacy of dietary and pharmacologic therapies in EoE is reflected not only by histologic remission but also by improvements in esophageal structure and function, including reduced need for dilation and increased esophageal caliber and distensibility [62,63,64]. In the pre-diagnostic era, a substantial proportion of patients required repeated dilations for fibrotic strictures, highlighting the importance of early anti-inflammatory treatment [63]. Longitudinal studies have shown that effective therapy can lead to gradual increases in esophageal diameter, even in the absence of dilation, suggesting partial reversibility of remodeling [65]. In addition, esophageal distensibility, assessed by techniques such as EndoFLIP, improves under anti-inflammatory treatment and correlates with symptom relief [66,67]. Early initiation of therapy may prevent fibrostenotic progression and reduce the need for repeated dilations, while combined approaches can provide both immediate and long-term benefits [68,69].

In the present case, dietary management was implemented through PEG-assisted enteral nutrition. This was a key component of the conservative strategy, as it allowed adequate nutritional support while minimizing esophageal mechanical stress and limiting exposure to potential food triggers during the healing phase. PEG-assisted nutritional support likely contributed substantially to closure of the fistulous tract.

Endoscopic dilation is the standard mechanical intervention in fibrostenotic EoE, indicated in patients with significant strictures or persistent dysphagia despite anti-inflammatory therapy [26,29,50,70,71]. Although control of inflammation is preferable before dilation, early intervention may be required in severe cases to restore luminal patency [72,73,74,75,76]. Dilation techniques, including bougie and balloon methods, have comparable efficacy and are performed gradually to achieve symptomatic improvement [50,77]. Dilation provides rapid relief of dysphagia but does not address the underlying inflammation, and recurrence is common in the absence of effective anti-inflammatory therapy [5,15,16,50,65,77,78,79,80,81,82,83]. Combined treatment strategies prolong remission, reduce the need for repeated procedures, and improve esophageal caliber and distensibility over time [66,84,85]. The procedure has a favorable safety profile when performed cautiously, with very low rates of perforation and serious complications [16,22,23,24,31,71,86,87]. Overall, dilation should be integrated into a comprehensive therapeutic approach aimed at achieving histologic remission and preventing fibrostenotic progression [16,26,50,84].

In this context, it is important to emphasize that endoscopic dilation treats exclusively the mechanical consequences of esophageal fibrosis and does not influence the underlying eosinophilic inflammation [72,73]. Accordingly, pharmacologic control of inflammation represents an essential element of the long-term management of eosinophilic esophagitis, playing a role both in preventing progression to fibrostenotic complications and in reducing the need for repeated endoscopic procedures [70].

Although eosinophilic esophagitis is predominantly managed with pharmacologic, dietary, and endoscopic therapies, surgical intervention may be required in rare, severe, or life-threatening scenarios, particularly when complications extend beyond the scope of conservative or minimally invasive management [88,89]. In the present patient, the absence of mediastinal involvement and clinical stability allowed a conservative, non-surgical approach.

Transmural perforation (Boerhaave-like) and mediastinal abscess represent the most severe complications, often requiring thoracotomy, drainage, and occasionally esophageal resection to control mediastinal contamination and prevent sepsis [90,91]. Conversely, partial ruptures or contained leaks can often be treated conservatively or through minimally invasive interventions, including covered esophageal stent placement combined with thoracoscopic mediastinal drainage, which has demonstrated full healing and rapid recovery without the need for open repair [92]. Recent case reports have also highlighted endoluminal vacuum therapy (EVAC) as a promising alternative for esophago-pleural fistula closure following spontaneous perforation, achieving successful outcomes without open surgery [93]. For patients presenting with diffuse fibrostenotic remodeling, recurrent strictures, or pseudodiverticular changes, surgical options such as segmental resection with primary anastomosis or esophageal reconstruction using gastric or colonic conduits may be considered in specialized centers, depending on the extent of structural damage [90]. Overall, surgery in EoE should be reserved for severe, life-threatening complications, such as full-thickness perforation or mediastinal abscess, while endoscopic and minimally invasive strategies (stenting, EVAC, dilation) remain the preferred approaches for selected cases within a multidisciplinary management framework [88,91,92]. In our patient, conservative management was appropriate because there was no evidence of mediastinal extension or clinical deterioration, and serial imaging and endoscopy demonstrated progressive resolution.

Eosinophilic esophagitis is a chronic inflammatory condition with fibrostenotic evolutionary potential, in which persistence of eosinophilic inflammation can lead not only to esophageal strictures and rings, but also, rarely, to atypical complications such as intramural collections or esophageal fistulas. The present case highlights the importance of correlating clinical, endoscopic, and histopathologic data to establish the correct diagnosis, particularly in unusual forms, as well as the need for rigorous differentiation from GERD. Most importantly, this case demonstrates that a blind-ending esophageal fistula complicating EoE may resolve with multidisciplinary conservative treatment, including anti-inflammatory therapy, PEG-assisted nutritional support, and close endoscopic follow-up, without the need for surgery in a carefully selected patient.

## 4. Conclusions

EoE is a chronic inflammatory condition with fibrostenotic evolutionary potential, and atypical complications, such as blind-ending esophageal fistula, remain rare but have a major impact on diagnostic and therapeutic pathways. Diagnosis relies on the correlation of the clinical and endoscopic findings with histologic confirmation, and the use of standardized instruments (such as EoEHSS) can refine the assessment of lesion severity and extent. Early and sustained control of inflammation is essential for preventing fibrotic remodeling and reducing the need for dilations.

From a therapeutic perspective, the optimal approach is multimodal and individualized: the combination of anti-inflammatory therapies (PPIs, topical steroids), dietary interventions, and selective endoscopic dilation for fixed strictures provides symptomatic improvement and long-term disease control. In refractory forms or those with a pronounced fibrostenotic phenotype, biologics, such as *dupilumab*, represent effective options. Multidisciplinary coordination (gastroenterology, general surgery, pathology, nutrition) and periodic endoscopic and histologic monitoring remain crucial to guide therapy, prevent recurrences, and optimize prognosis.

This case illustrates that a blind-ending esophageal fistula complicating EoE may resolve with multidisciplinary conservative management—including anti-inflammatory therapy and PEG-assisted nutritional support—without the need for surgery in carefully selected patients.

A conservative approach may be appropriate in selected cases of esophageal fistula complicating EoE, with multidisciplinary management being essential.

## Figures and Tables

**Table 1:** Eosinophilic Esophagitis Histology Scoring System (EoEHSS): histologic features and definitions Adapted from Collins et al. (2018) [13].

Feature	Definition
Eosinophilic inflammation	Based on peak eosinophil count
Basal zone hyperplasia	Basal zone occupies more than 15% of total epithelial thickness
Eosinophil abscess	Eosinophil aggregate that disrupts the underlying epithelial architecture
Eosinophil surface layering	Eosinophils align in one or more rows in the upper third of the epithelium
Dilated intercellular spaces	Intercellular bridges are visible in paracellular spaces
Surface epithelial alteration	Surface epithelial cells stain more darkly than normal and eosinophils that may be present among the altered epithelial cells
Dyskeratotic epithelial cells	Epithelial cells with deeply staining cytoplasm and shrunken hyperchromatic nuclei that generally occur singly and may be found anywhere in the epithelium
Lamina propria fibrosis	Coalesced fibrils form fibers of varying diameter

## Data Availability

The clinical data and investigations supporting the findings of this case report are included within the article. Additional raw data are not publicly available due to patient privacy and ethical restrictions. No new datasets were generated or analyzed for the review component.

## References

[1] Al-Horani R.A., Chiles R. (2022). First therapeutic approval for eosinophilic esophagitis. Gastroenterol. Insights.

[2] Yaxley J.P., Chakravarty B. (2015). Eosinophilic oesophagitis: A guide for primary care. Aust. Fam. Physician.

[3] Wąsik J., Małecka-Wojciesko E. (2023). Eosinophilic esophagitis-what do we know so far?. J. Clin. Med..

[4] Mona R., Hruz P. (2025). Epidemiology of eosinophilic esophagitis: Really a novel and evolving disease?. Inflamm. Intest. Dis..

[5] Miehlke S. (2015). Clinical features of eosinophilic esophagitis in children and adults. Best Pract. Res. Clin. Gastroenterol..

[6] Williams I., Taniere P., Goh J. (2012). Young man presenting with recurrent food bolus impaction. Clin. Med..

[7] Adamiak T., Plati K.F. (2018). Pediatric esophageal disorders: Diagnosis and treatment of reflux and eosinophilic esophagitis. Pediatr. Rev..

[8] Musburger B.G., Gonzalez Echeandia M., Suskind E.L., Suskind D.L., Zheng H.B., Mark D. (2025). Current and emerging therapies for eosinophilic esophagitis (EoE): A comprehensive review. Pharmaceutics.

[9] Uchida A.M., Burk C.M., Rothenberg M.E., Furuta G.T., Spergel J.M. (2023). Recent advances in the treatment of eosinophilic esophagitis. J. Allergy Clin. Immunol. Pract..

[10] Fernandez-Becker N.Q. (2021). Eosinophilic esophagitis: Incidence, diagnosis, management, and future directions. Gastroenterol. Clin. North Am..

[11] Odze R.D. (2009). Pathology of eosinophilic esophagitis: What the clinician needs to know. Am. J. Gastroenterol..

[12] Collins M.H., Martin L.J., Alexander E.S., Boyd J.T., Sheridan R., He H., Pentiuk S., Putnam P.E., Abonia J.P., Mukkada V.A. (2017). Newly developed and validated eosinophilic esophagitis histology scoring system and evidence that it outperforms peak eosinophil count for disease diagnosis and monitoring. Dis. Esophagus.

[13] Collins M.H., Capocelli K., Yang G.Y. (2018). Eosinophilic gastrointestinal disorders pathology. Front. Med..

[14] Younes M., Gui D. (2025). The pathologic diagnosis of eosinophilic esophagitis: Time for reassessment. Arch. Pathol. Lab. Med..

[15] Warners M.J., Oude Nijhuis R.A.B., de Wijkerslooth L.R.H., Smout A.J.P.M., Bredenoord A.J. (2018). The natural course of eosinophilic esophagitis and long-term consequences of undiagnosed disease in a large cohort. Am. J. Gastroenterol..

[16] Schoepfer A.M., Gonsalves N., Bussmann C., Conus S., Simon H.U., Straumann A., Hirano I. (2010). Esophageal dilation in eosinophilic esophagitis: Effectiveness, safety, and impact on the underlying inflammation. Am. J. Gastroenterol..

[17] Collins M.H. (2008). Histopathologic features of eosinophilic esophagitis. Gastrointest. Endosc. Clin. N. Am..

[18] Redd W.D., Barlowe T.S., LaFata S.S., Gee T.S., Thel H.L., Cameron B.A., Xue A.Z., Kiran A., Ocampo A.A., McCallen J. (2025). Differences in clinical presentation and treatment response of patients with eosinophilic esophagitis complicated by esophageal food impaction. Gastro Hep Adv..

[19] Hirano I., Aceves S.S. (2014). Clinical implications and pathogenesis of esophageal remodeling in eosinophilic esopha-gitis. Gastroenterol. Clin. N. Am..

[20] Chang F., Anderson S. (2008). Clinical and pathological features of eosinophilic oesophagitis: A review. Pathology.

[21] Michelon M., Savarino E.V., Montori M., Argenziano M.E., Poortmans P.J., Visaggi P., Penagini R., Tate D.J., Coletta M., Sorge A. (2025). Endoscopic dilation for fibrostenotic complications in eosinophilic esophagitis: A narrative review. Allergies.

[22] Arias-González L., Rodríguez-Alcolado L., Laserna-Mendieta E.J., Navarro P., Lucendo A.J., Grueso-Navarro E. (2024). Fibrous remodeling in eosinophilic esophagitis: Clinical facts and pathophysiological uncertainties. Int. J. Mol. Sci..

[23] Koutlas N.T., Dellon E.S. (2017). Progression from an inflammatory to a fibrostenotic phenotype in eosinophilic esoph-agitis. Case Rep. Gastroenterol..

[24] Sharma P., Yadlapati R. (2021). Pathophysiology and treatment options for gastroesophageal reflux disease: Looking beyond acid. Ann. N. Y. Acad. Sci..

[25] Gómez-Aldana A., Jaramillo-Santos M., Delgado A., Jaramillo C., Lúquez-Mindiola A. (2019). Eosinophilic esophagitis: Current concepts in diagnosis and treatment. World J. Gastroenterol..

[26] Greuter T., Safroneeva E., Bussmann C., Biedermann L., Vavricka S.R., Katzka D.A., Schoepfer A.M., Straumann A. (2019). Maintenance treatment of eosinophilic esophagitis with swallowed topical steroids alters disease course over a 5-year follow-up period in adult patients. Clin. Gastroenterol. Hepatol..

[27] Rajan J., Newbury R.O., Anilkumar A., Dohil R., Broide D.H., Aceves S.S. (2016). Long-term assessment of esophageal remodeling in patients with pediatric eosinophilic esophagitis treated with topical corticosteroids. J. Allergy Clin. Immunol..

[28] Lucendo A.J., Arias Á., Molina-Infante J. (2016). Efficacy of proton pump inhibitor drugs for inducing clinical and histologic remission in patients with symptomatic esophageal eosinophilia: A systematic review and meta-analysis. Clin. Gastroenterol. Hepatol..

[29] Beveridge C.A., Hermanns C., Thanawala S., Yang Q., Qin Y., Thota P.N., Hoscheit M., Brown J.M., Ivanov A.I., Lembo A. (2025). An esophageal luminal diameter of 16 mm predicts dysphagia resolution in eosinophilic esophagitis. Dig. Dis. Sci..

[30] Nennstiel S., Schlag C. (2020). Treatment of eosinophilic esophagitis with swallowed topical corticosteroids. World J. Gastroenterol..

[31] Hirano I., Chan E.S., Rank M.A., Sharaf R.N., Stollman N.H., Stukus D.R., Wang K., Greenhawt M., Falck-Ytter Y.T., Chachu K.A. (2020). AGA Institute and the Joint Task Force on Allergy-Immunology Practice Parameters clinical guidelines for the management of eosinophilic esophagitis. Gastroenterology.

[32] Papadopoulou A., Koletzko S., Heuschkel R., Dias J.A., Allen K.J., Murch S.H., Chong S., Gottrand F., Husby S., Lionetti P. (2014). Management guidelines of eosinophilic esophagitis in childhood. J. Pediatr. Gastroenterol. Nutr..

[33] Zhang X., Cheng E., Huo X., Yu C., Zhang Q., Pham T.H., Wang D.H., Spechler S.J., Souza R.F. (2012). Omeprazole blocks STAT6 binding to the eotaxin-3 promoter in eosinophilic esophagitis cells. PLoS ONE.

[34] Strauss A.L., Falk G.W. (2022). Refractory eosinophilic esophagitis: What to do when the patient has not responded to proton pump inhibitors, steroids and diet. Curr. Opin. Gastroenterol..

[35] Syverson E.P., Rubinstein E., Lee J.J., McDonald D.R., Hait E. (2024). The role of dupilumab in the treatment of eosinophilic esophagitis. Immunotherapy.

[36] Dellon E.S., Rothenberg M.E., Collins M.H., Hirano I., Chehade M., Bredenoord A.J., Lucendo A.J., Spergel J.M., Aceves S., Sun X. (2022). Dupilumab in adults and adolescents with eosinophilic esophagitis. N. Engl. J. Med..

[37] Chehade M., Dellon E.S., Spergel J.M., Collins M.H., Rothenberg M.E., Pesek R.D., Hirano I., Liu R., Laws E., Mortensen E. (2024). Dupilumab for eosinophilic esophagitis in patients 1 to 11 years of age. N. Engl. J. Med..

[38] Greuter T., Hirano I., Dellon E.S. (2020). Emerging therapies for eosinophilic esophagitis. J. Allergy Clin. Immunol..

[39] Leung J., Beukema K.R., Shen A.H. (2015). Allergic mechanisms of eosinophilic oesophagitis. Best Pract. Res. Clin. Gastroenterol..

[40] Straumann A., Conus S., Grzonka P., Kita H., Kephart G., Bussmann C., Beglinger C., Smith D.A., Patel J., Byrne M. (2010). Anti-interleukin-5 antibody treatment (mepolizumab) in active eosinophilic oesophagitis: A randomized, placebo-controlled, double-blind trial. Gut.

[41] Spergel J.M., Rothenberg M.E., Collins M.H., Furuta G.T., Markowitz J.E., Fuchs G., O’Gorman M.A., Abonia J.P., Young J., Henkel T. (2012). Reslizumab in children and adolescents with eosinophilic esophagitis: Results of a double-blind, randomized, placebo-controlled trial. J. Allergy Clin. Immunol..

[42] Youngblood B.A., Brock E.C., Leung J., Falahati R., Bochner B.S., Rasmussen H.S., Peterson K., Bebbington C., Tomasevic N. (2019). Siglec-8 antibody reduces eosinophils and mast cells in a transgenic mouse model of eosinophilic gastroenteritis. JCI Insight.

[43] Dellon E.S., Peterson K.A., Murray J.A., Falk G.W., Gonsalves N., Chehade M., Genta R.M., Leung J., Khoury P., Klion A.D. (2020). Anti-Siglec-8 antibody for eosinophilic gastritis and duodenitis. N. Engl. J. Med..

[44] Dellon E.S., Spergel J.M. (2023). Biologics in eosinophilic gastrointestinal diseases. Ann. Allergy Asthma Immunol..

[45] Ottoni M., Nicoletta F., Pederzani A., Barone A., Ridolo E. (2025). Personalization of therapy for patients with eosinophilic esophagitis. Expert Rev. Clin. Immunol..

[46] Dickerson A., Dellon E.S., Aceves S.S. (2025). Future of therapy and monitoring for eosinophilic esophagitis and eosinophilic gastrointestinal diseases. Ann. Allergy Asthma Immunol..

[47] Kalra S., Chowdhary R., Kalra E., Dhall S., Sohi G.S., Singh A., Vuthaluru A.R., Goyal M.K. (2025). JAK-STAT inhibitors: A game changer, from rheumatology to gastroenterology. Med. Res. Arch..

[48] Gautam R., Ruffner M.A. (2026). Eosinophilic esophagitis: Mechanisms of disease and approach to treatment. Curr. Allergy Asthma Rep..

[49] Herrera-deGuise C., Serra-Ruiz X., Lastiri E., Borruel N. (2023). JAK inhibitors: A new dawn for oral therapies in inflammatory bowel diseases. Front. Med..

[50] Pasta A., Calabrese F., Furnari M., Savarino E.V., Visaggi P., Bodini G., Formisano E., Zentilin P., Giannini E.G., Marabotto E. (2025). Endoscopic management of eosinophilic esophagitis: A narrative review on diagnosis and treatment. J. Clin. Med..

[51] Farah A., Assaf T., Hindy J., Abboud W., Mahamid M., Savarino E.V., Mari A. (2025). The dynamic evolution of eosinophilic esophagitis. Diagnostics.

[52] Arias A., González-Cervera J., Tenias J.M., Lucendo A.J. (2014). Efficacy of dietary interventions for inducing histologic remission in patients with eosinophilic esophagitis: A systematic review and meta-analysis. Gastroenterology.

[53] McGowan E.C., Platts-Mills T.A. (2016). Eosinophilic esophagitis from an allergy perspective: How to optimally pursue allergy testing and dietary modification in the adult population. Curr. Gastroenterol. Rep..

[54] Lucendo A.J., Arias Á., González-Cervera J., Yagüe-Compadre J.L., Guagnozzi D., Angueira T., Jiménez-Contreras S., González-Castillo S., Rodríguez-Domíngez B., De Rezende L.C. (2013). Empiric 6-food elimination diet induced and maintained prolonged remission in patients with adult eosinophilic esophagitis: A prospective study on the food cause of the disease. J. Allergy Clin. Immunol..

[55] Kagalwalla A.F., Sentongo T.A., Ritz S., Hess T., Nelson S.P., Emerick K.M., Melin-Aldana H., Li B. (2006). Effect of six-food elimination diet on clinical and histologic outcomes in eosinophilic esophagitis. Clin. Gastroenterol. Hepatol..

[56] Gonsalves N., Yang G.Y., Doerfler B., Ritz S., Ditto A.M., Hirano I. (2012). Elimination diet effectively treats eosinophilic esophagitis in adults; food reintroduction identifies causative factors. Gastroenterology.

[57] Molina-Infante J., Lucendo A.J. (2018). Dietary therapy for eosinophilic esophagitis. J. Allergy Clin. Immunol..

[58] Molina-Infante J., Arias Á., Alcedo J., Garcia-Romero R., Casabona-Frances S., Prieto-Garcia A., Modolell I., Gonzalez-Cordero P.L., Perez-Martinez I., Martin-Lorente J.L. (2018). Step-up empiric elimination diet for pediatric and adult eosinophilic esophagitis: The 2-4-6 study. J. Allergy Clin. Immunol..

[59] Rodríguez-Sánchez J., Gómez Torrijos E., López Viedma B., de la Santa Belda E., Martín Dávila F., García Rodríguez C., Brito F.F., Camacho J.O., Figueroa P.R., Molina-Infante J. (2014). Efficacy of IgE-targeted vs empiric six-food elimination diets for adult eosinophilic oesophagitis. Allergy.

[60] Chang J.W., Kliewer K., Haller E., Lynett A., Doerfler B., Katzka D.A., Peterson K.A., Dellon E.S., Gonsalves N., Aceves S.S. (2023). Development of a practical guide to implement and monitor diet therapy for eosinophilic esophagitis. Clin. Gastroenterol. Hepatol..

[61] Ketchum C.J., Strauss Starling A. (2025). Insights into the natural history and disease course of eosinophilic esophagitis. Ann. Allergy Asthma Immunol..

[62] Greenberg S., Chang N.C., Corder S.R., Reed C.C., Eluri S., Dellon E.S. (2021). Dilation-predominant approach versus routine care in patients with difficult-to-treat eosinophilic esophagitis: A retrospective comparison. Endoscopy.

[63] Schoepfer A.M., Safroneeva E., Bussmann C., Kuchen T., Portmann S., Simon H.U., Straumann A. (2013). Delay in diagnosis of eosinophilic esophagitis increases risk for stricture formation in a time-dependent manner. Gastroenterology.

[64] Sorge A., Masclee G.M.C., Bredenoord A.J. (2023). Endoscopic diagnosis and response evaluation in patients with eosinophilic esophagitis. Curr. Treat. Options Gastroenterol..

[65] Snyder D.L., Alexander J.A., Ravi K., Fidler J.L., Katzka D.A. (2024). Course of esophageal strictures in eosinophilic esophagitis using structured esophagram protocol. Gastro Hep Adv..

[66] Nicodème F., Hirano I., Chen J., Robinson K., Lin Z., Xiao Y., Gonsalves N., Kwasny M.J., Kahrilas P.J., Pandolfino J.E. (2013). Esophageal distensibility as a measure of disease severity in patients with eosinophilic esophagitis. Clin. Gastroenterol. Hepatol..

[67] Carlson D.A., Hirano I., Zalewski A., Gonsalves N., Lin Z., Pandolfino J.E. (2017). Improvement in esophageal distensibility in response to medical and diet therapy in eosinophilic esophagitis. Clin. Transl. Gastroenterol..

[68] Nhu Q.M., Aceves S.S. (2018). Medical and dietary management of eosinophilic esophagitis. Ann. Allergy Asthma Immunol..

[69] Khanna N. (2006). How do I dilate a benign esophageal stricture?. Can. J. Gastroenterol..

[70] Lucendo A.J., Molina-Infante J. (2018). Esophageal dilation in eosinophilic esophagitis: Risks, benefits, and when to do it. Curr. Opin. Gastroenterol..

[71] Chen C.X., Jin Z.A., Yang M., Tang F.T., Tang S.H. (2025). Endoscopic treatment of benign esophageal strictures: Advances and challenges. World J. Gastrointest. Surg..

[72] Runge T.M., Eluri S., Woosley J.T., Shaheen N.J., Dellon E.S. (2017). Control of inflammation decreases the need for subsequent esophageal dilation in patients with eosinophilic esophagitis. Dis. Esophagus.

[73] Runge T.M., Eluri S., Cotton C.C., Burk C.M., Woosley J.T., Shaheen N.J., Dellon E.S. (2016). Outcomes of esophageal dilation in eosinophilic esophagitis: Safety, efficacy, and persistence of the fibrostenotic phenotype. Am. J. Gastroenterol..

[74] Katzka D.A. (2019). Esophageal dilation as the primary treatment for eosinophilic esophagitis. Gastroenterol. Hepatol..

[75] Straumann A., Katzka D.A. (2018). Diagnosis and treatment of eosinophilic esophagitis. Gastroenterology.

[76] Aceves S.S., Alexander J.A., Baron T.H., Bredenoord A.J., Day L., Dellon E.S., Falk G.W., Furuta G.T., Gonsalves N., Hirano I. (2022). Endoscopic approach to eosino-philic esophagitis: American Society for Gastrointestinal Endoscopy consensus conference. Gastrointest. Endosc..

[77] Dougherty M., Runge T.M., Eluri S., Dellon E.S. (2017). Esophageal dilation with either bougie or balloon technique as a treatment for eosinophilic esophagitis: A systematic review and meta-analysis. Gastrointest. Endosc..

[78] Richter J.E. (2017). Esophageal dilation for eosinophilic esophagitis: It’s safe! Why aren’t we doing more dilations?. Gastrointest. Endosc..

[79] Jacobs J.W., Spechler S.J. (2010). A systematic review of the risk of perforation during esophageal dilation for patients with eosinophilic esophagitis. Dig. Dis. Sci..

[80] Muir A., Falk G.W. (2021). Eosinophilic esophagitis: A review. JAMA.

[81] Hagel A.F., Naegel A., Dauth W., Matzel K., Kessler H.P., Farnbacher M.J., Hohenberger W.M., Neurath M.F., Raithel M. (2013). Perforation during esophageal dilatation: A 10-year experience. J. Gastrointest. Liver Dis..

[82] Peterson K., Collins M.H., Aceves S.S., Chehade M., Gonsalves N. (2024). Concepts and controversies in eosinophilic esophagitis: What’s coming down the pipe?. Gastroenterology.

[83] Straumann A., Lucendo A.J., Miehlke S., Vieth M., Schlag C., Biedermann L., Vaquero C.S., de Los Rios C.C., Schmoecker C., Madisch A. (2020). Budesonide orodispersible tablets maintain remission in a randomized, placebo-controlled trial of patients with eosinophilic esophagitis. Gastroenterology.

[84] Shi P., Ding X. (2018). Progress on the prevention of esophageal stricture after endoscopic submucosal dissection. Gastroenterol. Res. Pract..

[85] Alexander J.A. (2014). Esophageal dilation in eosinophilic esophagitis. Tech. Gastrointest. Endosc..

[86] Moawad F.J., Molina-Infante J., Lucendo A.J., Cantrell S.E., Tmanova L., Douglas K.M. (2017). Systematic review with meta-analysis: Endoscopic dilation is highly effective and safe in children and adults with eosinophilic oesophagitis. Aliment. Pharmacol. Ther..

[87] Davis B.P., Rothenberg M.E. (2016). Mechanisms of disease of eosinophilic esophagitis. Annu. Rev. Pathol..

[88] Nasim R., Naseem H., Stammen M., Hoffman M., Mba B. (2020). Complications of eosinophilic esophagitis. Chest.

[89] Arias-González L., Rey-Iborra E., Ruiz-Ponce M., Laserna-Mendieta E.J., Arias Á., Lucendo A.J. (2020). Esophageal perforation in eosinophilic esophagitis: A systematic review on clinical presentation, management and outcomes. Dig. Liver Dis..

[90] Liacouras C.A., Furuta G.T., Hirano I., Atkins D., Attwood S.E., Bonis P.A., Burks A.W., Chehade M., Collins M.H., Dellon E.S. (2011). Eosinophilic esophagitis: Updated consensus recommendations for children and adults. J. Allergy Clin. Immunol..

[91] Gisasola P., Iriarte A., Larez M.R., Casanova L., Bujanda L. (2019). Mediastinal abscess, an unusual way of presentation of eosinophilic esophagitis. Allergy Asthma Clin. Immunol..

[92] Issa D., Alwatari Y., Smallfield G.B., Shah R.D. (2019). Spontaneous transmural perforation in eosinophilic esophagitis: Rare case presentation and role of esophageal stenting. J. Surg. Case Rep..

[93] Attwood S., Epstein J. (2021). Eosinophilic oesophagitis: Recent advances and practical management. Frontline Gastroenterol..

